# Characterization, isotherm, and thermodynamic data for selective adsorption of Cr(VI) from aqueous solution by Indonesia (Ende-Flores) natural zeolite Cr(VI)-imprinted-poly(4-VP-co-EGDMA)-ANZ (IIP-ANZ)

**DOI:** 10.1016/j.dib.2018.01.081

**Published:** 2018-02-05

**Authors:** Yantus A.B. Neolaka, Ganden Supriyanto, Handoko Darmokoesoemo, Heri Septya Kusuma

**Affiliations:** a Chemical Education Department, Faculty of Education and Teachers Training, University of Nusa Cendana, Kupang 85001, Nusa Tenggara Timur, Indonesia; b Department of Chemistry, Faculty of Science and Technology, Airlangga University, Mulyorejo, Surabaya 60115, Indonesia; c Department of Chemical Engineering, Faculty of Industrial Technology, Institut Teknologi Sepuluh Nopember, Surabaya 60111, Indonesia

**Keywords:** Natural zeolite, Imprinted-polymer, Selective adsorption, Hexavalent chromium

## Abstract

In this paper, we report for the first time modification of Indonesia (Ende-Flores) natural zeolite Cr(VI)-imprinted-poly(4-VP-co-EGDMA)-ANZ (IIP-ANZ) as a selective adsorbent for Cr(VI) from aqueous solution. The IIP-ANZ was synthesized from Cr(VI) as a template, 4-vinylphiridine (4-VP) as complex agent and as functional monomer, ethylene glycol dimethyl acrylate (EGDMA) as a cross-linker agent, benzoyl peroxide (BPO) as initiator and ethanol/acetone as a porogen. The optimization adsorption parameters optimization such as adsorbent amount, initial pH of sample solution, contact time and temperature were studied. The maximum adsorption capacity was 4.210 mg/g adsorbent. The adsorption process follow Freundlich isotherm model. Under the competitive condition, the adsorption capacity of IIP-ANZ for Cr(VI) is higher than Pb(II), Mn(II), NI(II) and Cr(III). Moreover, the reusability of the IIP-ANZ particle was tested for five times and no significant loss in adsorption capacity observed.

**Specifications Table**

**Table:** 

Subject area	*Chemical Engineering*
More specific subject area	*Adsorption*
Type of data	*Table, image, figure*
How data was acquired	– *The uptake of Cr(VI) by the adsorbent (q*_*e*_*) was determined based on the subtraction of the initial and final concentration of adsorbate* – *Fourier transform infrared (FTIR) spectroscopy (Shimadzu, IRPrestige 21), scanning electron microscopy with energy dispersive X-ray (SEM-EDX) spectroscopy (JEOL, JMS 5600, Tokyo, Japan), X-ray diffraction (Shimadzu, XRD-6000), Quantachrome Instruments NOVA 1200 (High-Speed Gas Sorption Analyzer Versions 10.0–10.03) was used for determine the characteristics of the adsorbent* – *The Cr(VI) concentration measurement was performed by UV–vis spectroscopy (Shimadzu, UV-1240)*
Data format	*Analyzed*
Experimental factors	– *To synthesize Indonesia (Ende-Flores) natural zeolite Cr(VI)-imprinted-poly(4-VP-co-EGDMA)-ANZ (IIP-ANZ), the Cr(VI) was used as a template, 4-vinyl pyridine (4-VP) was used as complex, functional monomer, ethylene glycol dimethacarylate (EGDMA) as a cross linker, benzoyl peroxide (BPO) as initiator and acid were activated of Indonesia (Ende-Flores) natural zeolite (ANZ) as a host* – *For comparison, NIP-ANZ (non-ion imprinted polymer) was also prepared using identical procedure without the addition of Cr(VI)* – *Data of IIP-ANZ were acquired for Cr(VI) removal from aqueous solution*
Experimental features	*IIP-ANZ for Cr(VI) adsorption from aqueous solution*
Data source location	*Airlangga University, Surabaya, Indonesia*
Data accessibility	*Data are accessible with the article*

**Value of the data**

• The newly synthesized adsorbent has a good potential application in related of wastewater treatment or to use in solid phase extraction
• The isotherm and thermodynamic data will be informative and useful for predicting and modeling the adsorption capacity and mechanism of chromium removal by the adsorbent
• The acquired data will be advantageous for the scientific community wanting to scale up and design an adsorption column with IIP-ANZ as medium for the removal of Cr(VI)-containing waters or wastewaters

## 1 Data

**Fig. 1:**
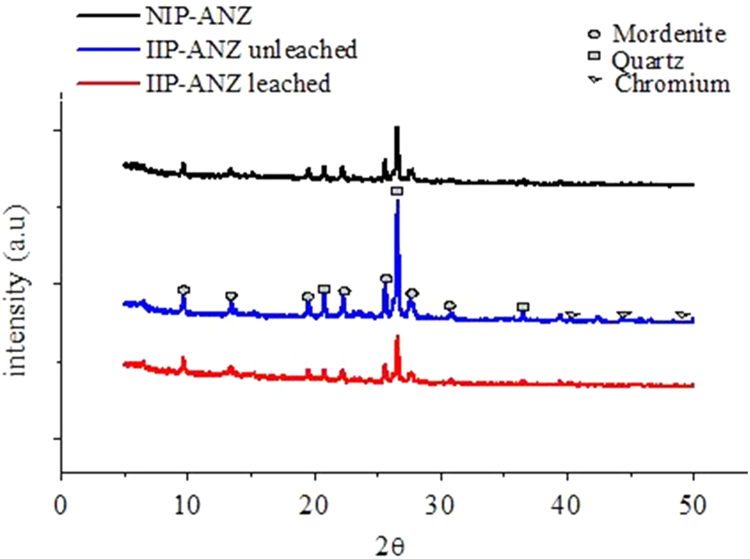
XRD patterns of IIP-ANZ unleached, IIP-ANZ leached and NIP-ANZ.

**Fig. 2:**
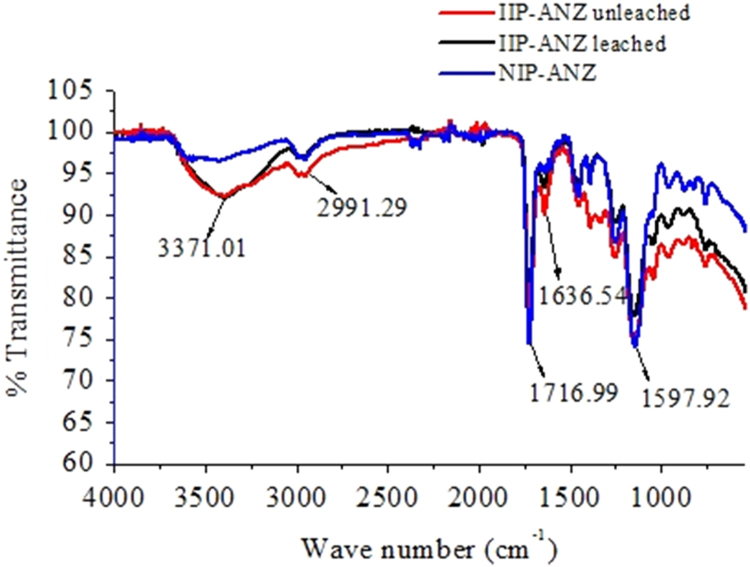
The FTIR Spectra of IIP-ANZ unleached, IIP-ANZ leached and NIP-ANZ.

**Fig. 3:**
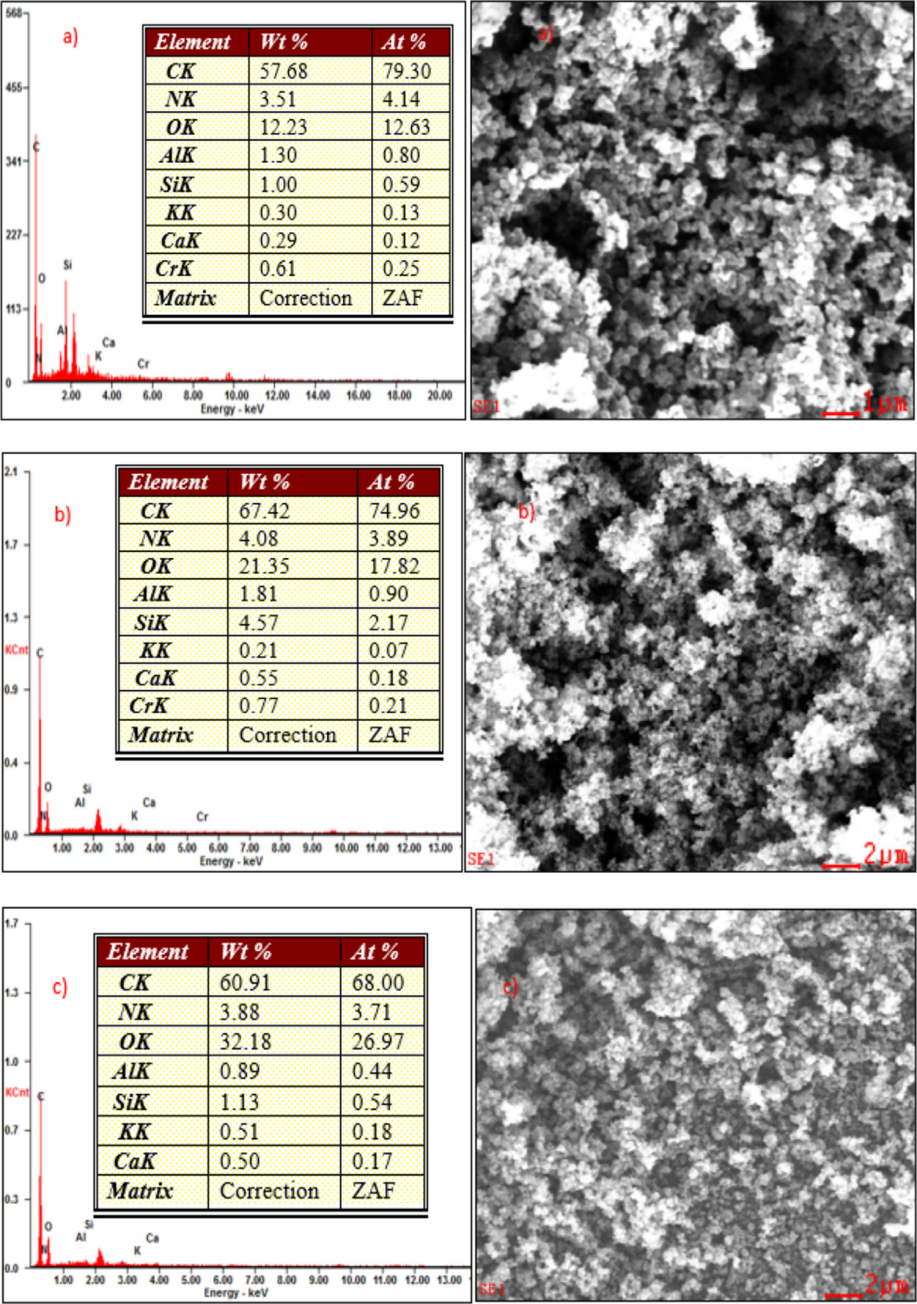
SEM-EDX analysis for: (a) IIP-ANZ unleached, (b) IIP-ANZ leached and (c) NIP-ANZ.

**Fig. 4:**
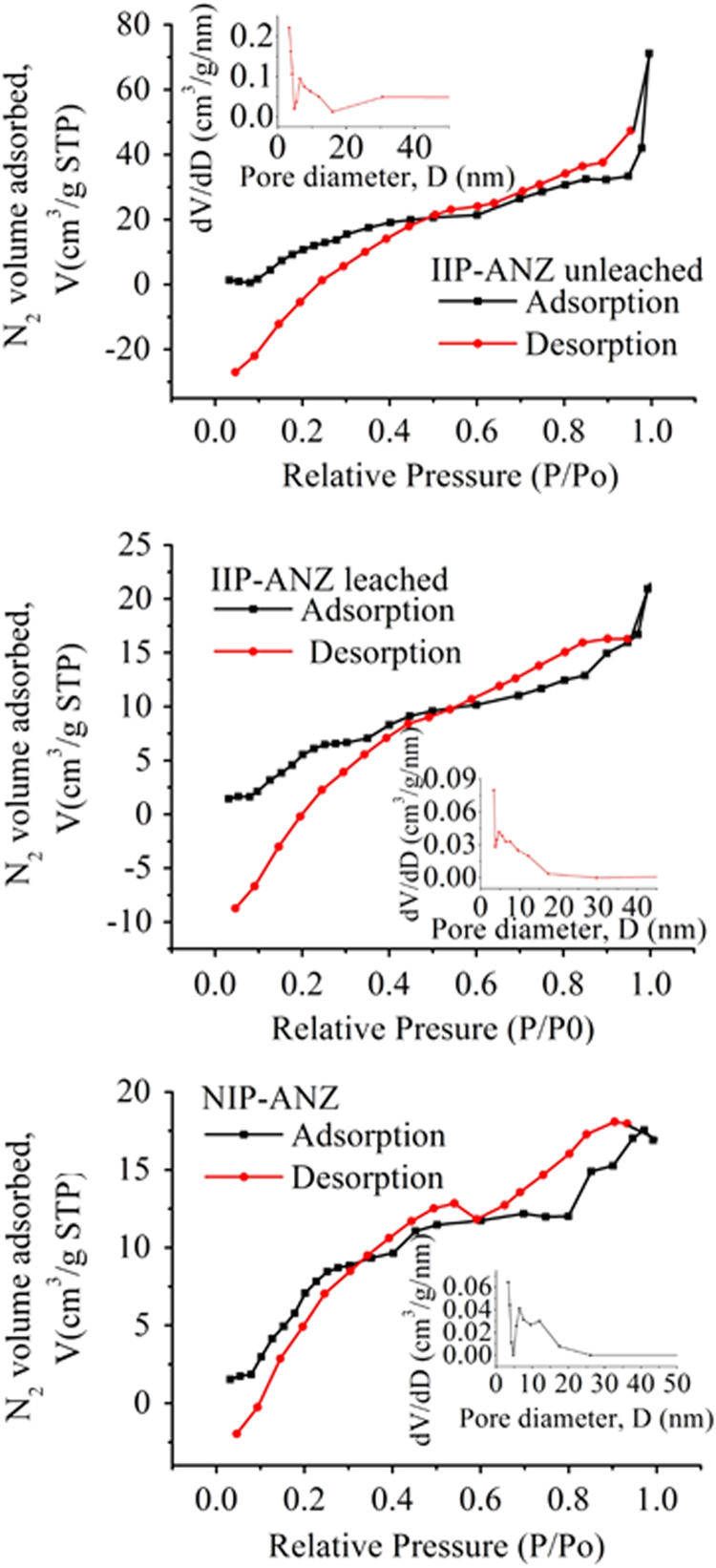
N_2_ adsorption isotherm and pore size distribution of IIP-ANZ unleached, IIP-ANZ leached and NIP-ANZ.

**Fig. 5:**
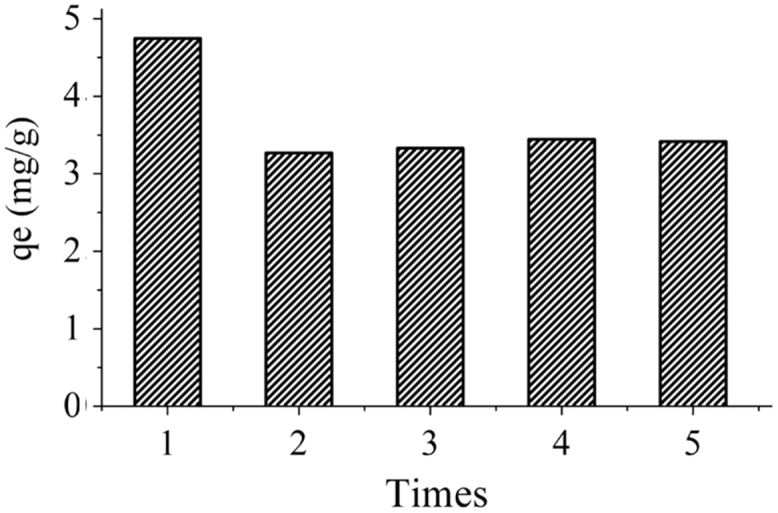
Reusability of IIP-ANZ.

The XRD patterns of IIP-ANZ unleached, IIP-ANZ leached and NIP-ANZ are shown in Fig. 1. The FTIR of IIP-ANZ unleached, IIP-ANZ leached and NIP-ANZ at wave numbers from 400 to 4000 cm^−1^ are given in Fig. 2. The results of the SEM-EDX analysis for IIP-ANZ unleached, IIP-ANZ leached and NIP-ANZ are shown in Fig. 3. N_2_ adsorption isotherm and pore size distribution of unleached IIP-ANZ unleached, IIP-ANZ leached and NIP ANZ was presented in Fig. 4 and Table 1. The pH of zero point charge, pH_ZPC_, for IIP-ANZ leached and NIP-ANZ obtained is shown in Table 1. The optimum condition for Cr(VI) adsorption on IIP-ANZ is presented in Table 2. The isotherm and thermodynamic parameters for the adsorption of Cr(VI) by IIP-ANZ and NIP-ANZ is presented in Table 3, Table 4. Adsorption capacities of IIP-ANZ and NIP-ANZ in the presence of competitive ions such as Cr(VI)/Pb(II), Cr(VI)/Mn(II), Cr(VI)/Ni(II) and Cr(VI)/Cr(III) was studied in a batch system and the result was presented in Table 5 and the calculated K_d_, k and k’ parameters are given in Table 6. the reusability of the IIP-ANZ, the adsorption-desorption cycle was repeated five times, and the results are shown in Fig. 5.

**Table 1:** Physical parameters of IIP-ANZ unleached, IIP-ANZ leached and NIP-ANZ.

Samples	BET surface area^a^ (m^2^/g)	Total pore volume^b^ (cm^3^/g)	Micropore volume^c^ (cm^3^/g)	Mesopore volume (cm^3^/g)	Average pore diameter (Å)	pH_ZPC_
IIP-ANZ unleached	142.268	0.0721	0.0000	0.0721	56.00	–
IIP-ANZ leached	146.697	0.2205	0.1612	0.0593	39.12	2.24
NIP-ANZ	54.456	0.0261	0.003	0.0231	65.21	4.96

a Multi point BET.

b Total volume pore total at P/P0=0.99893 (IIP-ANZ unleached), 0.99319 (IIP-ANZ leached) and 0.99197 (NIP-ANZ).

c Mesopore volume=Total pore volume – Micropore volume.

**Table 2:** Optimum condition for Cr(VI) adsorption on IIP-ANZ (The concentration of Cr(VI) solution is 14 mg/L).

Parameters	Optimum value	q_e_ (mg/g)	Adsorption efficiency (%)
Adsorbent amount (g)	0.08	2.862	65.42
pH	2.00	4.373	99.96
Time (min)	30.00	6.386	72.99
Temperature (K)	323	3.220	36.80
Average value		4.210	68.79

**Table 3:** Isotherm parameters for the adsorption of Cr(VI) by IIP-ANZ and NIP-ANZ.

Isotherm adsorption models	Parameters	Adsorbents	
		IIP-ANZ	NIP-ANZ
Langmuir	Qmax (mg/g)	0.176	0.956
	K_L_ (L/mg)	−1.960	−1.376
	R^2^	0.259	0.3203
Freundlich	n	0.637	2.382
	K_F_ (mg/kg)	0.010	1.165
	R^2^	0.725	0.6121

**Table 4:** Results of thermodynamic experiment for adsorption Cr(VI) onto IIP-ANZ and NIP-ANZ.

T (K)	∆G^o^ (kJ/mol)		∆H^o^ (kJ/mol)	
	IIP-ANZ	NIP-ANZ	IIP-ANZ	NIP-ANZ
303	−228.846	−14.537	−120.162	−126.639
313	−232.432	−10.837	∆S^o^ (kJ/mol)	
323	−236.019	−7.138	IIP-ANZ	NIP-ANZ
333	−239.606	−3.438	0.359	−0.370
343	−243.193	0.262

**Table 5:** Competitive adsorption of Cr(VI)/Pb(II), Cr(VI)/Mn(II), Cr(VI)/Ni(II) and Cr(VI)/Cr(III) on the IIP-ANZ and NIP-ANZ.

Ion		q_e_ (mg/g)	
		IIP-ANZ	NIP-ANZ
Cr(VI)/Pb(II)	Cr(VI)	2.921	0.197
	Pb(II)	0.273	0.038
Cr(VI)/Mn(II)	Cr(VI)	4.678	2.441
	Mn(II)	0.504	1.127
Cr(VI)/Ni(II)	Cr(VI)	7.625	7.280
	Ni(II)	1.829	1.847
Cr(VI)/Cr(III)	Cr(VI)	3.741	2.978
	Cr(III)	1.035	1.291

**Table 6:** The distribution coefficient (Kd), selectivity coefficient (k) and relative selectivity coefficient (k’) for IIP-ANZ and NIP-ANZ.

Ions		IIP-ANZ		NIP-ANZ		k′
		K_d_ (L/g)	k	K_d_ (L/g)	k	
Cr(VI)/Pb(II)	Cr(VI)	0.313	–	0.197	–	–
	Pb(II)	0.022	14.199	0.038	5.184	2.73
Cr(VI)/Mn(II)	Cr(VI)	0.718	–	0.242	–	–
	Mn(II)	0.062	11.532	0.159	1.524	7.564
Cr(VI)/Ni(II)	Cr(VI)	4.239	–	3.094	–	–
	Ni(II)	4.127	1.027	4.447	0.696	1.476
Cr(VI)/Cr(III)	Cr(VI)	0.467	–	0.322	–	–
	Cr(III)	0.441	1.058	0.668	0.483	2.191

## 2 Experimental design, materials and methods

### 2.1 Reagents and materials

Sodium hydroxide, 1,5-diphenyl carbazide, potassium dichromate, sulphuric acid, hydrochloric acid, acetone, nitric acid, NH_4_Cl, CrCl_3_. 6H_2_O, Ni_2_SO_4_, Mn_2_SO_4_, Pb(NO_3_)_2_, 4-vinyl pyridine (4-VP), ethylene glycol dimethacarylate (EGDMA), benzoyl peroxide (BPO), acid were activated of Indonesia (Ende-Flores) natural zeolite (ANZ) which was used during the whole experiment. All reagents used in this research were purchased from Merck (Singapore) and Sigma Aldrich (Singapore).

### 2.2 Synthesis of IIP-ANZ and NIP-ANZ

To synthesize IIP-ANZ, the Cr(VI) was used as a template, 4-VP was used as complex, functional monomer, EGDMA as a cross linker, BPO as initiator and ANZ as a host. IIP-ANZ were synthesized directly through precipitation method in which 4-VP (12 mmol; 13 mL) and Cr(VI) (1 mmol; 0.3 g) were sealed in a polymerization bottle (250 mL) and added to ethanol and acetone with proportion ethanol: acetone (2:1). This solution was kept at room temperature for 30 minutes to form metal-complex 4-VP-Cr(VI). After this period, EGDMA (60 mmol; 11.3 mL), 1% BOP (0.1 g in 10 mL chloroform) and ANZ (10 g) was slowly dropped into the polymerization bottle containing 4-VP-Cr(VI) complex. The mixture was purged with nitrogen gas for ten minutes, close a glass bottle and polymerization in water bath thermostatic at 65 °C for one hour then raised the temperature to 80 °C and kept constant for five hours. After polymerization, the solid polymer was filtered and stirred in ethanol: demineralization water (70: 30) for 6 hours in order to remove the excess of the reagents. The imprint anion (Cr(VI)) was removed by stirring solid polymer in 4 M HNO_3_ for 6 h. The solid polymer was filtered through 0.45 μm filter paper and a fresh nitric acid solution was added, the process was continued until the optimized amount of removed Cr(VI) was achieved and determined by UV–vis spectrophotometer. The solid polymer was then collected and washed several times with demineralization water until reached neutral pH. The solid polymer was dried at 55 °C and being observed. For comparison, NIP-ANZ (non-ion imprinted polymer) was also prepared using identical procedure without the addition of Cr(VI).

### 2.3 Optimization parameters of sorption studies

Sorption of metal ions from aqueous solutions was investigated in batch experiments. Optimization of pH was studied from pH 1 to pH 9. The pH of the Cr(VI) solution was adjusted by 0.1 M HCl or 0.1 M NaOH. Ideal weight adsorbent was optimized from 0.01 g to 2.0 g. Optimization of time adsorption was investigated from 0 minutes to 120 min. All optimization parameters were performed with 50 mL of Cr(VI) solution 14 mg/L. Optimization of temperature was performed from 303 to 343 K by using all optimum conditions, namely various concentrations of Cr(VI) from 6 mg/L to 14 mg/L by adding the suspension solution in close glass flask and stirrer at constant rpm for each temperature. To determine Cr(VI) concentration in bulk sample, the solutions was filtered and added with 2.0 mL diphenyl carbazide in solution, mix and add H_2_SO_4_ solution to give a pH of 2 ± 0.5, dilute to 100 mL with aqua demineralization and let stand 5 to 10 min for full colour development and measure its with UV–vis spectrophotometer at 540 nm. Some metal ions sorbed onto the unit mass of adsorbent was calculated from Eq. (1).

(1) $$q_{e}=\frac{\left(C_{0}-C_{e}\right)V}{m}$$

where C_o_ is the initial concentration of Cr(VI) in solution (mg/L), C_e_ is the equilibrium concentration (mg/L), q_e_ is the equilibrium adsorption capacity (mg/g), m is the mass of adsorbent (g), and V is the volume of solution (L).

The removal percentage of Cr(VI) can be calculated by the following equation.

(2) $$\mathrm{Efficiency}\;\mathrm{of}\;\mathrm{adsorption}\left(\%\right)\;=\frac{C_{0}-C_{e}}{C_{0}}x100$$

where C_o_ is the initial concentration of Cr(VI) in solution (mg/L) and C_e_ is the equilibrium concentration (mg/L).

### 2.4 Analysis and characterization

The phases of NIP-ANZ and IIP-ANZ was characterized by X-ray diffraction using PANalytical, X’pert Pro. For determination the funcional groups on the adsorbent surface a Fourier transform infrared (FTIR) spectrometer (Shimadzu, IRPrestige 21) was applied. The morphological surface and elemental composition of the adsorbent was examined using scanning electron microscopy with energy dispersive X-ray (SEM-EDX) spectroscopy (JEOL, JMS 5600, Tokyo, Japan). The surface area, total pore volume, and pore size distribution were determined using Quantachrome Instruments NOVA 1200 (High-Speed Gas Sorption Analyzer Versions 10.0–10.03). The pH_ZPC_ and pH was measured using Pasco pH meter (spark PS-2008A) and the Cr(VI) ion before and after adsorption with IIP-ANZ or NIP-ANZ was analyzed spectrophotometrically (Shimadzu UV-1240) at 540 nm using 1,5-diphenyl carbazide as the complexing agent. Other while total chromium, Ni(II), Mn(II), Pb(II) ion were analyzed using AA500 atomic absorption spectrometer made in PG instruments.

### 2.5 Isotherm adsorption studies

Two model isotherm adsorption was used in this experimental such as Langmuir isotherm and Freundlich isotherm. The Langmuir isotherm is usually utilized for a monolayer adsorption at specific homogeneous sites on Cr(VI)-4-VP-ANZ ion imprinted polymer or Cr(VI)-4-VP-ANZ non ion imprinted polymer surface. Eq. (3) is the Langmuir expression.

(3) $$\frac{C_{e}}{q_{e}}=\frac{1}{K_{L}q_{\max}}+\frac{1}{q_{\max}}C_{e}$$

where q_max_ is monolayer sorption capacity (mg/g) and K_L_ is Langmuir equilibrium constant (L/g) [1]. A plot of C_e_/q_e_ versus C_e_ allows determining the Langmuir constants. Freundlich isotherm is the earliest known relationship describing the non-ideal and reversible adsorption, not restricted to the formation of a monolayer [2]. This empirical model can be applied to multilayer adsorption, with non-uniform distribution of adsorption heat and affinities over the heterogeneous surface [2]. Eq. (4) is the Freundlich expression [1].

(4) $$\log\;q_{e}=\log K_{F}+\frac{1}{n}\log C_{e}$$

where *K*_F_ and *n* are Freundlich constants, the *K*_F_ is adsorption capacity while n is sorption intensity; q_e_ is amount of Cr(VI) per unit mass of adsorbate (mg/g); C_e_ is equilibrium concentration (mg/L). The plot of q_e_ versus C_e_ allows determining the Freundlich constants.

### 2.6 Thermodynamic study

Varying concentration of Cr(VI): 6, 8, 10, 12 and 14 mg/L at optimum condition of pH, adsorbent dosage, contact time and the temperature was used to find out the thermodynamics parameter of Cr(VI) adsorption on IIP-ANZ or NIP-ANZ. The standard enthalpy change (∆H°) and the standard entropy (∆S°) for Cr(VI) sorption on IIP-ANZ or NIP-ANZ were obtained using Van’t Hoff equation.

(5) $$\ln K_{L}=\frac{{∆H}^{o}}{\mathrm{RT}}+\frac{{∆S}^{o}}{R}$$

where *K*_L_ is the adsorption coefficient from the Langmuir adsorption isotherm, ∆H° is the standard enthalpy change (J/mol), ∆S° is the standard entropy change (J/mol/K), R is the gas constant (8.314 J/mol/K) and *T* is the temperature in *K*. The plot of ln *K*_L_ versus 1/*T* allows determining the standard enthalpy change (∆H°) and the standard entropy (∆S°).

The standard Gibbs free energy change (∆G°) of adsorption was calculated from Eq. (6) [3].

(6) $${∆G}^{o}={∆H}^{o}\;-\;{T∆S}^{o}$$

### 2.7 Selectivity studies

To study the competitive adsorption between the target and competing ion can use the distribution coefficient equation (Eq. (7)) [4].

(7) $$K_{d}=\frac{C_{i}-C_{f}}{C_{f}}$$

where *K*_d_, *C*_i_ and *C*_f_ represent the distribution coefficient, initial concentration and final solution concentration (mg/L). *V* and *m* are the volumes of the solution (L) and mass of the IIP-ANZ or NIP-ANZ (g). Selectivity coefficient for binding of ion target in present of an ion competitor can use the Eq. (8).

(8) $$k=\frac{K_{d}\;(\mathrm{template metal})}{K_{d}\left(\mathrm{interferent metal}\right)}$$

where k is the selectivity coefficient of interfering metal (i.e., Pb(II) ions). A comparison of the k values of the imprinted polymer with those of metal ions allows an estimation of the effect of imprinting on selectivity. To evaluate an imprinting effect, a relative selectivity coefficient (k’) was defined as follows Eq. (9) [4], [5].

(9) $$k^{\prime }=\frac{k(\mathrm{imprinted})}{k(\mathrm{non}-\mathrm{imprinted})}$$

where k’ is the indicator of the effect of imprinting on the selectivity of Cr(VI) adsorption on IIP-ANZ.
